# Comparing the performance of functional versus taxonomic metagenomics for detecting ammonia disturbances in the biogas system

**DOI:** 10.1093/femsec/fiag029

**Published:** 2026-03-20

**Authors:** Dries Boers, Olivier Chapleur, Anders F Andersson, Anna Schnürer

**Affiliations:** Department of Molecular Sciences, Swedish University of Agriculture (SLU), 750 07 Uppsala, Sweden; PROSE, National Research Institute for Agriculture, Food and Environment (INRAE), Paris-Saclay University, 92761 Antony Cedex, France; Department of Gene Technology, Science for Life Laboratory, KTH Royal Institute of Technology, 100 44 Stockholm, Sweden; Department of Molecular Sciences, Swedish University of Agriculture (SLU), 750 07 Uppsala, Sweden

**Keywords:** anaerobic digestion, biogas, ammonia disturbance, metagenomics, orthology, classification

## Abstract

Biogas is a renewable energy source with great potential, but its production is frequently hindered by process disturbances, of which a high ammonia concentration is one common cause. It is desirable that such disturbances are found as early as possible; metagenomics data has the potential to improve this detection. This study compares functional and taxonomic aspects of metagenomics data, hypothesizing that functional data will perform better for detecting ammonia disturbances. The hypothesis was tested by metagenomic sequencing of samples from three independent studies, which followed lab-scale reactors during ammonia disturbances. The resulting sequences were used to predict genes, which were functionally and taxonomically annotated. The read counts of these features were fitted to disturbance states and ammonia concentrations of reactor samples using regularized regression, which allowed filtering out irrelevant features even with limited sample sizes. Within studies, taxonomic data had similar or better performance in detecting ammonia disturbances and in fitting ammonia concentrations. When applying trained models to other studies however, while performance was generally poor, functional models more often performed better compared to taxonomic models than the other way around. All in all, our hypothesis that functional metagenomics would outperform taxonomic metagenomics only found limited support.

## Teaser

Biogas production is often hindered by disturbances. This study attempted to predict such disturbances using microbial DNA. Contrary to expectations, a functional perspective did not consistently outperform a taxonomic perspective.

## Introduction

### The biogas system and ammonia disturbances

Biogas is an energy source which can be produced carbon-neutrally while handling society’s biodegradable waste streams. Its purified form, biomethane, can replace fossil methane gas and anaerobic digestion, the process in which biogas is produced, allows for the recycling of the nutrients (nitrogen, phosphorous) in the input waste streams (Farghali et al. 2022). These properties make biogas an attractive energy source, and the European Union has dedicated itself to upscaling its production of biogas in the REPowerEU plan (European Commission 2022).

A challenge in biogas production is that the process is sensitive to disturbances, which lead to reduced biogas production, and in severe cases even process failure (Wu et al. 2019). Such disturbances have been shown to occur frequently, lasting from weeks up to months, with methane output decreasing by 30% (Nielsen and Angelidaki 2008). As a preventive measure, most biogas plants are being operated at lower organic loading rate than needed for reaching optimal biogas production (Wu et al. 2019).

A common cause for disturbances of the anaerobic digestion process is ammonia. Anaerobic digestion consists of interlocking chains of microbial metabolism. The final link in this network are the methanogens, methane-producing archaea, of which some are particularly sensitive to ammonia poisoning (Jiang et al. 2019). The cellular mechanism of this inhibition is not clear, but the ability of ‘free’ ammonia (NH_3_) to permeate across membranes seems to be involved, because toxicity is lower at lower pH, at which more ammonia is converted to ammonium ions (NH_4_^+^), which are not freely-diffusing due to their charge (Gallert et al. 1998). When the methanogens are inhibited, this leads to the accumulation of upstream metabolites, such as hydrogen and carbon dioxide, and also acetate and other volatile fatty acids (VFAs) (Rajagopal et al. 2013). Such build-ups are therefore clear signals of a disturbance.

### Towards microbial community monitoring

Because of the cost of disturbances in biogas plants, the process parameters of anaerobic digestion are often intensively monitored. In a review of such monitoring, it was shown that VFAs and biogas composition are indicators of disturbances at an early stage (Wu et al. 2019). However, it has been suggested that including data on the microbial community could improve monitoring effectiveness (Ferguson et al. 2018, Singh 2021), as such data might reveal disturbances at an even earlier stage than only chemical parameters (Singh 2021), and because models based upon chemical parameters may stop working after large microbiological shifts (Lemaigre et al. 2018).

The state of a microbial community can be assessed by analyzing its DNA. Two widely-used techniques are available for this: amplicon sequencing, which targets specific taxonomic marker sequences, and whole-genome ‘shotgun’ sequencing, which randomly samples sequences from all genomes in a sample. This study mainly concerns itself with data generated by the latter technique, and refers to it as metagenomic data. After sequence processing, such data can be analyzed from two perspectives. First, the taxonomic perspective views ‘who’ is present in a sample, that is, which taxa are present. Second, the functional perspective regards which genetic functions are present and which metabolic pathways are encoded. The two alternative perspectives raise the question which of them reflects the state of a microbial system best.

### Comparing the taxonomic and functional perspectives

Intuitively, the functional perspective looks more promising than the taxonomic perspective, as functions could build more fine-grained models of the biogas system. This intuition is further supported upon the concept of functional redundancy, in which taxa can be replaced by other taxa with identical functions without any effect on the system as a whole (Carballa et al. 2015). Furthermore, microbial traits can vary greatly between closely-related organisms, even within species (Welch et al. 2002). A phenomenon that also supports the functional perspective is that traits can be transmitted to distant taxa in horizontal gene-transfer, although it has been proposed that such transfer mostly concerns simple traits such as antibiotic resistance, while complex traits such as methanogenesis are strongly coupled to taxonomy (Martiny et al. 2015).

Findings of whether the functional or taxonomic perspective is superior have varied. In the general microbiological discussion, Xu et al. (2014) showed that in the Human Microbiome Project, the difference in classification accuracy using taxonomic 16S (rRNA gene) amplicon sequences and metagenomic functional annotation was not significant. However, in a landmark paper of the microbial biogeography field, Louca et al. (2016) showed that in the invariable micro-environments of rain-forest beaker plants, functional profiles predicted from 16S amplicon data were more constant than the taxonomic data itself, which led them to suggest that functional data in their field should be ‘the baseline […], particularly when the ultimate focus is on ecosystem functioning’. Again in a medical context, Casimiro-Soriguer et al. (2022) compared the use of functional and taxonomic profiles to predict colorectal cancer based upon fecal metagenomes from seven independent studies, finding that taxonomic data generally performed best. In summary, whether functional or taxonomic features give better performance is still ‘subject to debate’ (Hernández Medina et al. 2022).

Even though many studies have investigated the microbial composition and activity in biogas systems, the functional perspective has rarely been compared to the taxonomic perspective. For example, Campanaro et al. (2018) looked at taxonomy and function of the assembled metagenomes of different full-scale biogas plants, but focused on correlation of taxonomy to process parameters. In another study, Fischer et al. (2019) followed inoculates in batch experiments, and in half of these ammonia was increased. However, while 16S amplicon profiles and transcriptomics were both analyzed, their information content was not compared. A study by Lin et al. (2023) may be the most informative for the question in mind; they followed nine reactors over time to study the predictability of response to changes in feeding. They found that this change was both reflected in an altered taxonomy composition and in (functional) metatranscriptomic data. However, they did not compare which of these perspectives followed the change best.

### Aim and hypothesis

In this study, we aim to assess whether functional metagenomic data is more accurate than taxonomic data in representing the state of microbiological systems, specifically in the case of biogas reactors which are disturbed due to changing ammonia concentrations. The goal of this study is to contribute to the function versus taxonomy debate described above, both in general and in the specific context of the biogas system. Our hypothesis is that functional analysis will perform better than taxonomic analysis. To emphasise the confirmatory approach of this study, the hypothesis has been ‘preregistered’ (Wagenmakers et al. 2012) in a document which was digitally signed before starting with data analysis (Supplementary Data - File 1). The study was further expanded to include a taxonomic perspective based upon 16S amplicon data. Amplicon data is widely used, and is generally less expensive to generate than shotgun sequencing data, so including a comparison of the two types is of general interest.

By testing this hypothesis within the biogas system, we intend to explore the possibility for improving its monitoring, both by reassessing the performance of the taxonomy perspective, which has been used in biogas research more frequently, and by including the functional perspective, which is less explored.

## Methods

### Sample selection and classification

Three independent studies were identified which all followed ammonia-induced disturbances over time in multiple lab-scale, mesophilic (37°C) semi-continuous stirred-tank reactors. These studies are referred to as the Lemaigre study (Lemaigre et al. 2018), the Cardona study (Cardona et al. 2022) and the Ahrens study (Ahrens et al. 2025), and their characteristics are summarized in Table 1.

**Table 1:** Description of included studies.

Study (year)	Number of reactors	Hydraulic retention time	Substrate	Ammonia source	pH range	Ammonia concentration maximum (FAN, g/l)	Produced methane range (l/day)
Lemaigre et al. (2018)	3 (1)^a^	56 days	Beet pulp	Urea	6.8–8.52(6.29–7.641)^a^	4.07(0.0267)^a^	0.00–65.29(17.60–51.79)^a^
Cardona et al. (2022)	6	25 days	Food waste	Ammonium-carbonate	7.76–8.98	2.02	0.00–0.45
Ahrens et al. (2025)	5	53 days	Manure, grass, wheat flour and bran	Protein	7.51–8.18	0.471	1.42–4.12

a. Values in parentheses concern control reactor.

For each of these studies, reactor performance parameters over time were analyzed; these included feeding parameters, gas production, free ammonia nitrogen (FAN) concentrations, VFA concentrations and 16S amplicon profiles. Based upon this analysis, a selection for metagenomics sequencing was made of available samples. The selected samples were also classified into ‘disturbed’ and ‘undisturbed’ classes based upon the same parameters; especially their VFA concentrations. DNA was extracted from the selected samples using protocols that differed between the studies. For the Lemaigre study, new extractions were made using the AllPrep PowerViral DNA/RNA Kit (QIAGEN, NL). For the Cardona study, DNA was used that had been extracted for their original study using the PowerSoil DNA Isolation Kit (MO BIO, USA) and which had been stored at −80°C. For the Ahrens study, extractions were made using the FastDNA Spin Kit for Soil (MP Biomedicals, USA). The resulting DNA extracts were sent to Eurofins Genomics (LU) for sequencing (Illumina Novaseq, paired end, >10 M reads per sample, 150 bp per read). The resulting raw sequencing reads have been deposited in the European Nucleotide Archive (see ‘Data availability’).

### Sequence processing

**Figure 1:**
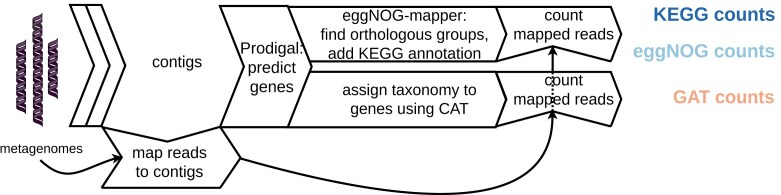
Workflow diagram of the shotgun metagenomics data processing to functional counts (in blue) and taxonomic counts (in light-red). GAT: gene-level annotation of taxonomy.

The resulting sequences were processed using the Snakemake metagenomics workflow nbis-meta (Sund 2021). An adapted version of this workflow can be found on Zenodo and GitHub (see Data availability), in which versions, options and parameters are provided for all tools; a visual summary is provided in Fig. 1. The workflow started with standard read processing; that is, trimming using Trimmomatic (Bolger et al. 2014), quality-checking using FastQC (Babraham Bioinformatics 2010) and MultiQC (Ewels et al. 2016), assembly into contigs (each sample individually) using Megahit (Li et al. 2015), read alignment to their respective assembly using Bowtie 2 (Langmead and Salzberg 2012), and duplicate removal using SAMtools (Danecek et al. 2021).

The further processing of contigs is described in greater detail, as their functional and taxonomic annotation is of specific interest to this publication. Protein-coding genes were predicted within contigs by Prodigal (Hyatt et al. 2010). For functional annotation, eggNOG-mapper 2 (Cantalapiedra et al. 2021) mapped the amino acid sequences of the predicted genes to the eggNOG database version 5 (Huerta-Cepas et al. 2019, eggNOG 2021), using DIAMOND (Buchfink et al. 2021) for alignment, and then used this alignment to assign eggNOG orthologous groups (eggNOG_OGs). eggNOG was used because it is the largest database for orthologs; it is automatically inferred based upon evolutionary patterns. Its size decreases the risk of missing functions of understudied micro-organisms, which have been suggested to be abundant in the biogas system (Campanaro et al. 2020). Besides the assigned eggNOG_OG, KEGG orthology data (Kyoto Encyclopedia of Genes and Genomes, 2025) included from the eggNOG database was used for additional functional annotation levels.

For taxonomic annotation, predicted protein-coding gene amino acid sequences were aligned by CAT (Von Meijenfeldt et al. 2019), also using DIAMOND, against the NCBI non-redundant protein database (National Center for Biotechnology Information 2021a), after which the last common ancestor of the matches with a low E-value and large sequence similarity was determined based upon the NCBI taxonomy database (National Center for Biotechnology Information, 2021b). It should be noted that the original implementation of CAT used a majority vote per contig, which is not included in this analysis; therefore, the current procedure is referred to as ‘gene-level annotation of taxonomy’ (GAT). Finally, functional and taxonomic features were quantified by counting the reads mapping to every protein-coding gene predicted by Prodigal and summing these per feature. The annotated features and gene counts have been deposited in Zenodo (see Data availability).

To include 16S amplicon data in the comparison, sequencing counts were included from the original studies. These sequencing counts were based upon different sequencing protocols and also on different methods for inferring sequence clusters (using amplicon sequence variants or operational taxonomic units) and different taxonomy databases.

Importantly, 16S and GAT data types contain a hierarchical component, that is, organisms which differ at species rank may be the same when viewed at domain rank. A similar hierarchy holds for KEGG, because in that database, counts can be analyzed per ortholog (the lowest level), per module, or pathway level (the highest level).

### Statistical analysis

**Figure 2:**
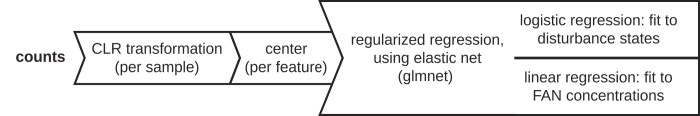
Workflow diagram of the statistical analysis of feature counts. CLR: centered log-ratio transformation. FAN: free ammonia nitrogen.

Statistical analysis of the functional and taxonomic feature counts was performed in R (R Core Team 2024) using packages from the tidyverse (Wickham et al. 2019). An exemplary analysis can be found in Supplementary Data - File 2, in which versions for all packages are included. Furthermore, code for performing all steps of the analysis has been deposited in Zenodo and on GitHub (see Data availability). See Fig. 2 for a visual summary of the analysis workflow.

First, the read counts for all features were transformed per sample, using the centered log ratio from package vegan (Oksanen et al. 2022), to compensate for a compositionality bias (Gloor et al. 2017). Principal components (PCs) of the transformed feature count data were then computed using the prcomp function (R Core Team 2024), and the scores of a PC were multiplied by its eigenvalue to visualize the explained variance of each component. To only generate one PC for each of the 16S, GAT and functional data types, the data before rank selection was used (OTU or ASV in 16S, ‘as is’ in GAT, and eggNOG).

Subsequently, regularized regression was applied to the transformed feature counts. Regularization makes large and complex models smaller and simpler by removing features, which is necessary when the number of features (far) outnumbers the number of samples in a dataset. The package glmnet (Friedman et al. 2010) was used, and elastic net models were constructed using mixing parameter α = $\frac{1}{2}$. The elastic net uses the regularization parameter ‘λ’ to apply lasso-like penalties based upon the number of variables, resulting in the removal of features that affect model performance least, and uses the same λ to apply ridge-like penalties based upon the sum of squared coefficients. The combination of the two types of penalties circumvents the problem of solution instability due to multicollinearity, which is incurred when only applying lasso penalization (Zou and Hastie 2005). An advantage of using elastic net in this study is that this approach does not allow for the modelling of non-linear effects. This limitation decreases the risk of overfitting the dataset, which is relevant when working with limited numbers of samples.

Two distinct types of regularized regression were applied. In logistic regularized regression, feature data was fit to the disturbed versus undisturbed classification. In linear regularized regression, feature data was fit to FAN concentrations, which are the primary cause of the disturbance of these reactors. Both of these data types had added value, because while the classification dataset can integrate multiple data types in a simple, explainable way, so-called separation can make the performance of models with more features than samples meaningless (Mansournia et al. 2018). Linear regression is not hindered by the same phenomenon.

For every value of λ, models were trained on datasets from the sample data of all but one reactor. For validation, the respective ‘out-reactor datasets’ were then fit to these trained models. This strategy was chosen over leave-one-out cross-validation, because the performance of the latter could be inflated by validation samples of a reactor matching the test sample profile of samples of the same reactor. In validation, so-called loss functions were used to negatively quantify the performance of the regularized models for their comparison. The defaults for logistic and linear regression were used: deviance and mean squared error (MSE), respectively. The mean loss value of models was plotted against the number of features of a model. The number of features is inversely related to λ, because as models become more regularized, their number of features decreases. Models with minimal mean loss and, secondarily, a minimal number of included features were selected from the fitted models. In linear regression, such minimal mean loss models could be selected directly, while in logistic regression, loss tends to decrease as models grow larger, and therefore the smallest models with near-minimal loss were selected manually instead. In data types with multiple hierarchical levels, these minimal loss models were used to determine and select the best-performing subtype. In the case of multiple levels sharing a similar optimal performance, a preference was given to higher levels because this might give better performance in subsequent cross-study testing. However, the highest taxonomic levels domain, superkingdom and kingdom were not selected, because their small number of features would likely lead to overfitting in regularized regression.

After selecting the best-performing count subtypes, the functional and taxonomic count types were compared to one another. The performance of the minimal loss models of the selected types was assessed on test sets from its own type from all studies. For example, the KEGG module’s minimal loss model for logistic regression trained on the Lemaigre study, was tested for the prediction of disturbance states of samples from the Cardona study, based upon that study’s KEGG module counts. Besides deviance and MSE, performance metrics included the area under the receiver operating characteristic curve (AUC) for logistic regression and the coefficient of determination (R^2^) for linear regression. These two metrics are both useful for comparing between studies, because both have a fixed range between 0 and 1. Finally, to get more insight in the regularized models, the features of the minimal loss models were extracted.

## Results

### Sample selection and classification

**Figure 3:**
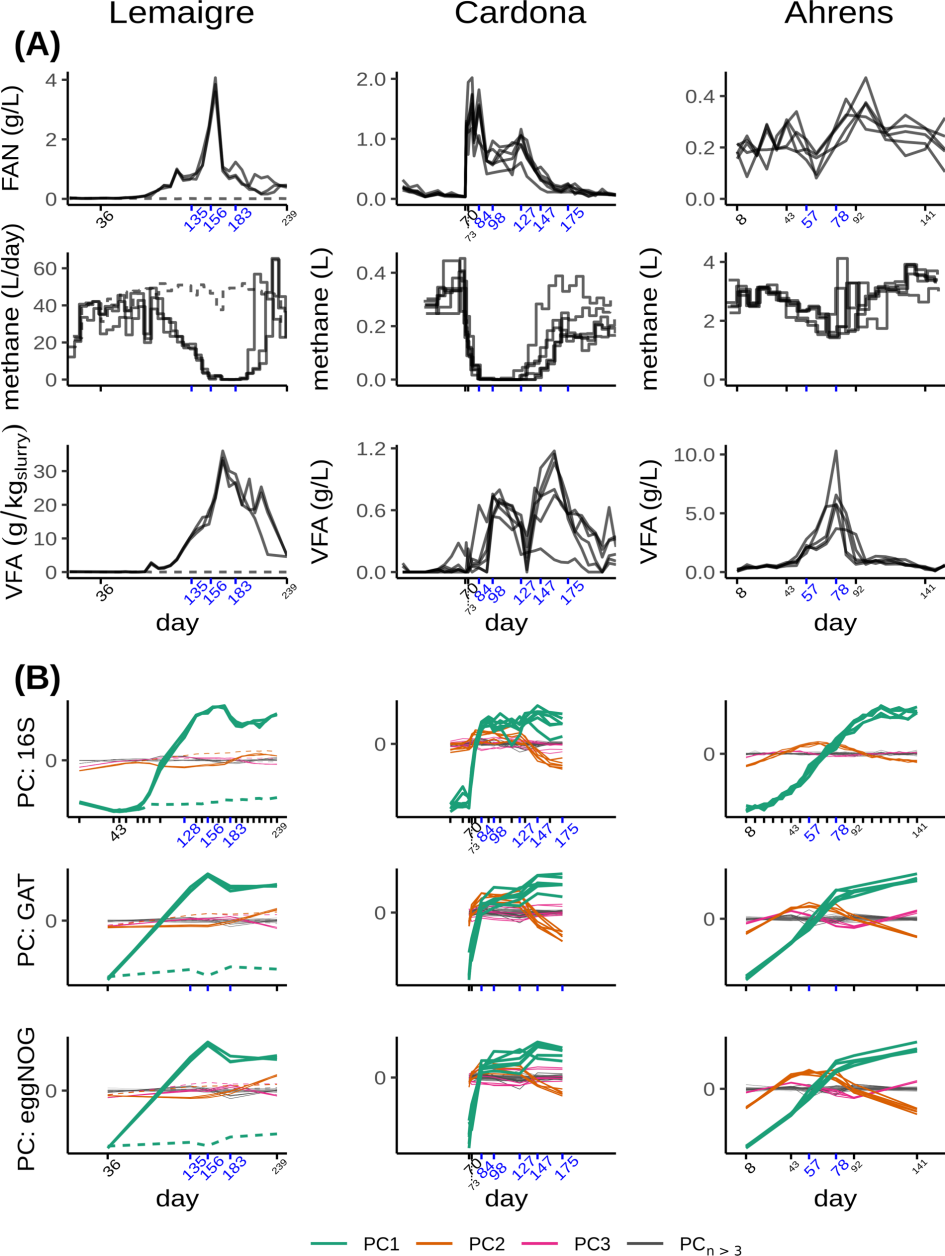
Reactor parameters (A) and principal components (B) over time for all three studies. Labeled points on time-axis indicate metagenomic samples (or alternative samples used for 16S sequencing). Blue labels indicate time points classified as disturbed, black labels with large digits indicate undisturbed time points, and black labels with small digits indicate unclassified time points. In Lemaigre panels, dashed line represents control reactor without addition of extra ammonium. In PC plots (B), component scores have been multiplied by their respective eigenvalues to accentuate the first PCs, which explain most variance. The range of these multiplied scores is uninformative, therefore only the zero-point is indicated. The 16S count dataset from which PCs were computed included additional time points, represented as axis ticks without label. 16S: 16S rRNA gene. FAN: free ammonia nitrogen. GAT: gene-level annotation of taxonomy. PC: principal component. VFA: volatile fatty acid.

Reactor parameters were collected from three independent studies, in which biogas reactors encountering ammonia disturbances were followed over time. Three of these parameters are presented in Fig. 3A, and the feeding parameters in Suppl. Table 1. In all three studies, concentrations of FAN changed over time in all reactors (except for the control reactor of the Lemaigre study). For the Lemaigre and Cardona studies, the changes were induced as part of their study design, while for the Ahrens study, this occurred due to high protein content in the substrate. Coincidentally with the changes in the FAN concentrations, a disturbance occurred, that is, the production of methane decreased, and VFA concentrations increased. The disturbances were followed by recovery events with methane production returning to initial values and VFA concentrations decreasing. The magnitude of VFA concentration peaks varied between the studies, but VFA accumulation was in all cases coupled to a decreased methane production, illustrating the imbalance in the microbial community metabolism. The duration of the disturbance, as predicted based on the VFA levels, varied between the studies. Expressed in hydraulic retention time (HRT), which quantifies how long a volume remains in a reactor, the Ahrens study showed the fastest recovery time (∼50 days, 1 HRT), while the Lemaigre and Cardona studies took longer to recover (both at least 100 days, which respectively corresponded to 2 and 4 HRTs). In the Ahrens study, the relative levels of change of the reactors’ FAN levels were also much smaller than those of the other studies, and their methane production never fully halted.

**Figure 4:**
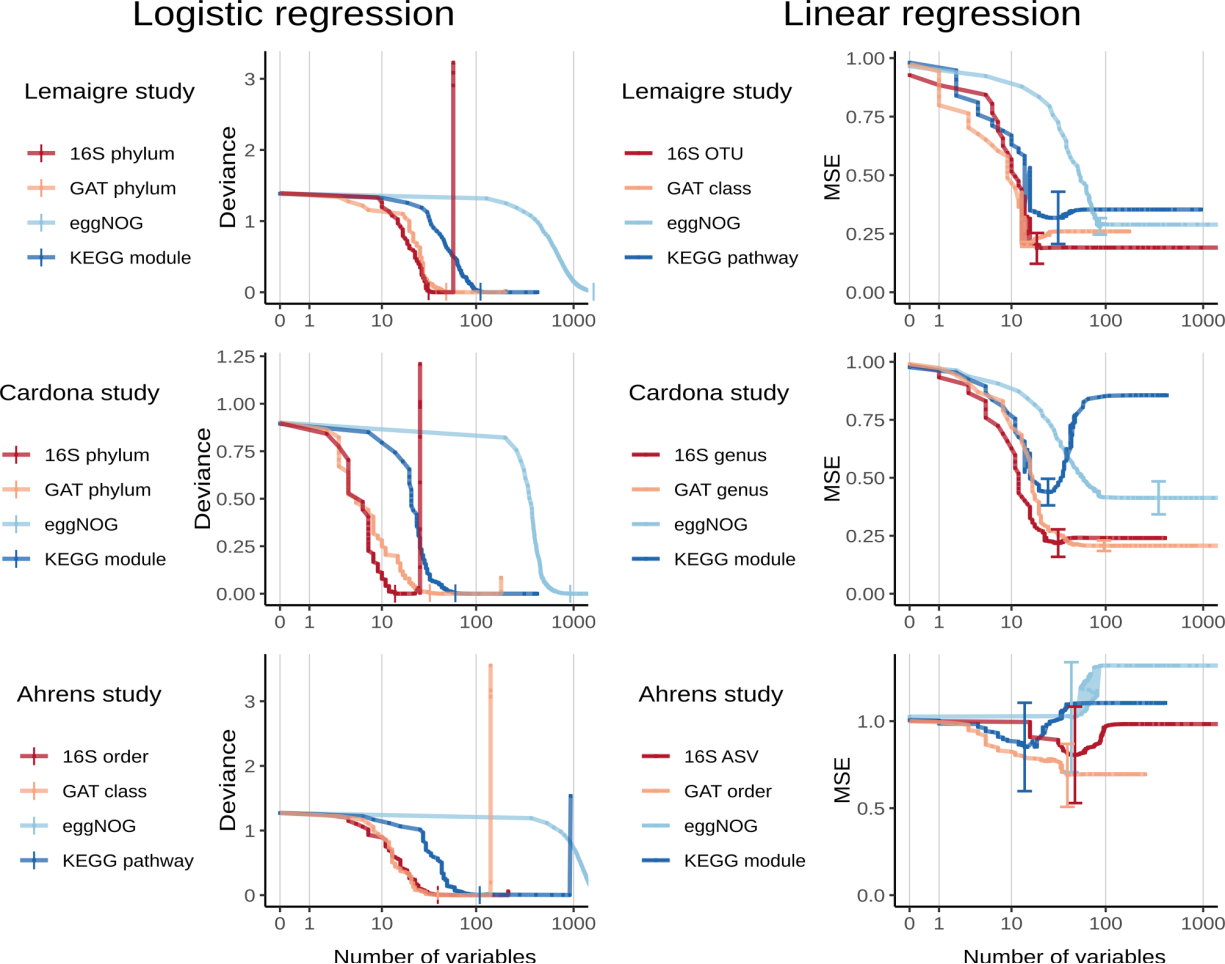
Comparison of regularized logistic and linear regression models, which are based upon functional or taxonomic count data, across studies. Models’ mean loss values, calculated by cross-validation, are plotted against the number of included variables, on a logarithmic scale. As loss function, deviance is used for logistic regression and MSE for linear regression. In logistic regression, smallest models with near-minimal mean loss (per count type, per study) are marked with a vertical bar. In linear regression, standard error bars have been added to models with minimal mean loss. 16S: 16S rRNA gene. ASV: amplicon sequence variant. GAT: gene-level annotation of taxonomy. MSE: mean squared error. OTU: Operational Taxonomic Unit.

Selected time points for the Lemaigre and Cardona studies consisted of time points at the ends of phases of their feeding regimes as described in their publications, which generally matched VFA profiles. For the Cardona study, day 73 and 147 were also added, because in a principal component analysis based upon 16S data (Fig. 4 of that study (Cardona et al. 2022)) these samples fell outside the three sample clusters to which (end-of-feeding-phase time points) day 70, 98, 127, and 175 belong. For the Ahrens study, time points for metagenomic sequencing were selected based upon VFA concentration profiles.

The classification of all time points as disturbed or undisturbed (as indicated in Fig. 3A) was primarily based upon VFA concentrations for all studies. Day 73 of the Cardona study and day 43 and 92 of the Ahrens study were left unclassified however, and left out of further analysis, as their status was considered unclear. The same was the case for day 239 of the Lemaigre study and 141 of the Ahrens study, because they were taken after disturbance, which neither corresponded to the undisturbed state, nor to the disturbed state.

### Unsupervised analysis: principal component analysis

The metagenomic sequencing data was processed without problem, so that the mean sample proportion of reads mapping to predicted assembled genes ranged from 61.0%–67.2% over all studies (see Suppl. Table 2 for standard deviations and read survival percentages after earlier steps). The annotation of predicted genes combined with the mapping resulted in functional and taxonomic count data. These metagenomic counts were combined with taxonomic 16S amplicon count data. As an initial exploratory analysis to see how the count data aligned with the reactor parameters and imputed disturbance states over time, PCs were computed and plotted (Fig. 3B). For every study, PC1 looked very similar between data types, and the same was true for PC2. This was also true for 16S data; the (subtle) differences of its PC curves compared to the metagenomic data types were likely caused by the fact that the 16S data consisted of more samples.

In the Lemaigre and Cardona study, PC1 (of all data types) fitted the disturbance states well. However, in the Ahrens study, PC1 was already increasing from the first sampling point, which made it difficult to distinguish changes caused by the disturbance from effects due to initial stabilization of the reactors. In that study, PC2 instead seemed to follow the VFA concentration more closely.

### Supervised analysis: regularized regression

The different annotation variants of count data were used to build models. Logistic regression models were used to predict samples’ disturbance status and linear regression models were used to estimate FAN concentrations, which was considered to be the principal cause of the disturbance.

For both types of regression analysis, each dataset was analyzed at multiple, hierarchical levels. For example, metagenomes could be annotated at different taxonomic ranks when using the GAT tool; the same was true for 16S rRNA sequences. When annotating a metagenome functionally using the KEGG database, this could be done at the lowest, single ortholog level, but also on a higher, module or pathway level. For functional and taxonomic count types, the percentages of counts annotated at different levels is summarized in Suppl. Table 3. Interestingly, while the taxonomic highest level 'GAT' had a larger count percentage annotated than the functional highest level 'eggNOG', the taxonomic lowest level 'GAT species' had a lower count percentage annotated than the lowest functional level 'KEGG KO'.

For each count type, per study, the performances in regularized logistic regression and linear regression per hierarchy level (Suppl. Figs. S1-S3) were compared to determine an optimal level (listed in Suppl. Table 4). A first group of observations concerned the best-performing hierarchy levels of taxonomic count types. These were always (12/12 cases) of lower rank in linear regression than in logistic regression. The difference in rank was large: while the majority (4/6 cases) of best-performing levels in logistic regression was phylum rank, a same share of linear regression was rank genus or lower. Second, regarding the functional data types, eggNOG consistently performed worst in logistic regression, and KEGG KO the second worst. In linear regression however, eggNOG’s minimal mean loss was lower than that of the KEGG data types in two studies, albeit within the latter’s standard error ranges and with very large models. Furthermore, KEGG KO never performed best in linear regression, but in all studies was in the standard error range of the best-performing KEGG type. A third class of observations regarded model sizes: there was no clear difference in number of features of best-performing models for every count type at every level between functional and taxonomic count types in general, but best-performing eggNOG models were largest in all studies and regression types.

The optimal hierarchy levels for all count types are included in Fig. 4, where their logistic and linear regression performance are compared within studies. Taxonomic count types (16S and GAT) generally had better performance than the functional count types (KEGG and eggNOG), in both logistic regression and linear regression. That is, for the same number of variables, logistic and linear regression respectively had similar or lower mean deviance and mean MSE (both of which are loss functions, which are lower for better-fitting models). While model deviance approximated zero in all count types in logistic regression as model size increased, indicating that all count types could perfectly predict disturbance state, this low-deviance stage was reached with fewer variables by taxonomic count types.

It is also noteworthy that in most comparisons, 16S data had near-identical performance as GAT data. The exceptions to this observation were the logistic regression comparison of the Cardona study, where a 16S model reached perfect performance with fewer variables than the GAT model (of identical rank) and the linear regression comparison of the Ahrens study, where the best GAT model had better mean performance than the 16S model with best mean performance (although standard error ranges overlapped).

For every study, regularized logistic and linear models with minimal (or near-minimal) loss were selected. These ‘trained’ models were then applied to data from all studies to test their cross-study performance (of which metrics are shown in Table 2, while own-study performance is shown in Suppl. Table 5). The 16S count type was omitted from this analysis due to different indexing databases in different studies. When first comparing own-study performance, it is remarkable that trained logistic regression models had good performance scores (high AUC; low deviance), but in specific cases the trained linear regression models performed worse (lower R^2^; higher MSE) on own datasets: this was the case for the KEGG module type of the Cardona study, and the GAT order and eggNOG types of the Ahrens study.

**Table 2:** Cross-study performance of regularized regression models.

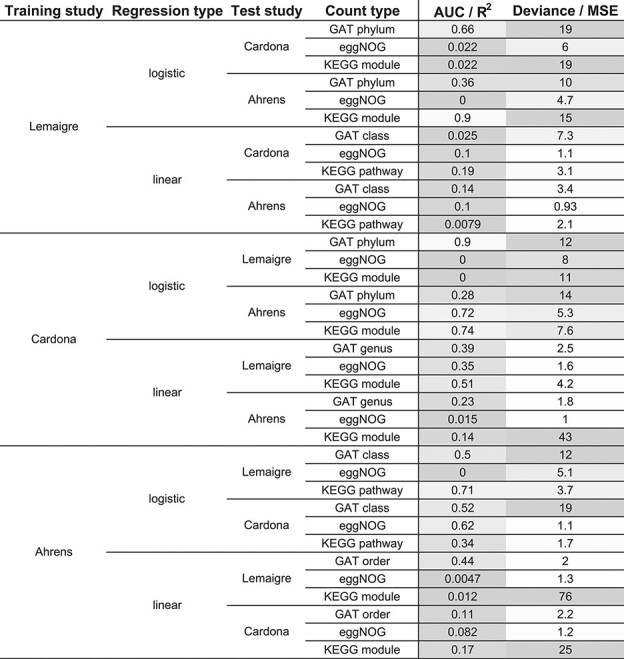

The ‘AUC / R^2^ ’ and ‘Deviance / MSE’ columns represent AUC and Deviance for logistic regression and R^2^ and MSE for linear regression, respectively. Cell shading is relative: for ‘AUC / R^2^’ all values are compared, while ‘Deviance / MSE’ values are compared within the same test study and regression type. AUC: area under the receiver operating characteristic curve. GAT: gene-level annotation of taxonomy. MSE: mean squared error. R^2^: the coefficient of determination.

The cross-study performance of models was generally poor, but this performance varied greatly. Logistic models’ mean AUC was 0.362 (standard deviation (SD): 0.366) and mean deviance was 9.68 (SD: 5.84); linear models’ mean R^2^ was 0.212 (SD: 0.225) and mean MSE was 9.99 (SD: 19.7). A better-performing exception was the Lemaigre study’s logistic regression model trained on KEGG module counts, when tested on the Ahrens study. Another exception was the Cardona study’s logistic regression model trained on GAT phylum counts, when tested on the Lemaigre study. For linear regression models, the main exception to the general observation of poor performance were the models trained on the Cardona study which were applied to the Lemaigre study. These models had three of the four top-ranking R^2^ values (excluding own-study testing).

When the performances of functional count types eggNOG and KEGG were compared to that of the taxonomic count type GAT, one or more functional count types had both higher AUC/R^2^ and lower Deviance/MSE for 3/6 test datasets (excluding same-study data) in logistic regression and 1/6 test sets in linear regression. The reverse, taxonomic count types outperforming functional count types, was not observed in any of the twelve combinations of regression types and test sets. No differences in performance were found between the three studies.

Finally, the features of all minimal loss models were extracted, together with their regression coefficients. Although an in-depth description of all included features is beyond the scope of this study, the number of features shared across regression types, from the same studies, are listed in Suppl. Table 6. The greatest overlap of features between logistic and linear regularized regression models was observed in the Cardona study, while the Lemaigre and Ahrens study had a lower degree of feature sharing. The overlap of regression types was greater for taxonomic count types than for the functional count type eggNOG. When comparing features shared between studies using the same regression algorithm (Suppl. Table 7) only few features were shared in linear regression. In logistic regression, the overlap of regression types was again greater for 16S and GAT than for eggNOG.

## Discussion

Given the interest in including microbial community data in biogas monitoring, we have compared the value of functional and taxonomic information for detecting process disturbances in reactors. In an initial unsupervised analysis of 16S rRNA counts, taxonomic GAT and functional eggNOG counts, main PCs of functional and taxonomic count data looked similar (Fig. 3). Subsequent regularized regression analyses (Fig. 4) showed that models based on taxonomic parameters could achieve a similar or better fit than models based on functional parameters, and this was the case in three independent lab-scale studies of ammonia disturbance. Furthermore, extracted features of the best-performing regularized regression models showed a greater overlap for taxonomic features than for functional features (Suppl. Tables 6-7), which would suggest greater stability. However, while the performance of trained models on other studies was generally poor (Table 2), models using eggNOG and KEGG counts fitted test study data better than GAT models in multiple combinations of studies and regression types, while the reverse (GAT models having a better fit) was not observed. Nonetheless, in sum, the results do not convincingly support our hypothesis that functional metagenomics data represent the biogas process better than taxonomic metagenomics data.

When comparing our results to findings reported in earlier literature, they do not match the statement by Louca et al. (2016) that functional data should be the ‘baseline’ of future microbial studies (they only claimed this for the context of biogeography however). Even in linear regression, taxonomic models gave a similar or better fit for FAN concentrations, which does not support the expectation that functional models, which are more fine-grained, would perform better. On the other hand, our results do match the statistically insignificant difference in classification accuracy between 16S amplicon data and metagenomic annotation found by Xu et al. (2014), as well as the findings of Casimiro-Soriguer et al. (2022), who found taxonomic data outperforming functional data in a human microbiome context.

The presented results do not support the hypothesized fine-grained nature of functional models, as functional models did not consistently have more features than taxonomic models. While eggNOG type models did have a larger size, these are not as convincing as KEGG models would be, because their annotation has a taxonomic component. The concept of functional redundancy (Carballa et al. 2015) was supported in cross-study validation by functional count data outperforming taxonomic count data more often than the other way around, but this result was undermined by greater feature sharing between taxonomic models.

To explain the underwhelming performance of the functional data types, one could hypothesize that functional databases are even more incomplete than taxonomic databases. The biogas system includes many poorly-described microorganisms (Campanaro et al. 2020). For these, the functional annotation of orthologs is likely to be based upon experiments in distantly-related organisms, which could reduce the reliability of these annotations. However, this reasoning is not in line with the finding that fewer counts were annotated at the GAT species level than at the KEGG KO level (Suppl. Table 3).

### rRNA amplicons, metagenomic DNA, and metatranscriptomic RNA

The finding that between taxonomic data types, the metagenomic GAT counts did not consistently outperform counts based upon 16S rRNA data is in line with recent results in human microbiome research, where metagenomic data has been found to yield ‘similar performances’ or has performed only ‘slightly better’ (Bars-Cortina et al. 2024). This implies that for current environmental microbiological analyses, 16S rRNA amplicon sequencing-based techniques may suffice, or even be preferable because of their lower cost. The promise of such 16S rRNA-based monitoring has been supported in a meta-analysis (Cortez-Cervantes et al. 2024). However, the performance of metagenomic data might be improved by using the full CAT workflow (Von Meijenfeldt et al. 2019). CAT includes a majority vote for all genes on a contigs, and this vote was excluded here for the sake of comparison to functional data, which is always analyzed per gene. Furthermore, cross-study testing of 16S data was not possible in the current study, because different workflows were used, including different extraction protocols and 16S rRNA regions for sequencing, and different software and databases for analysis. To allow for an improved comparison between studies, the first (easier) step would be to use the same analytic pipeline and database for 16S read processing. As a follow-up, an attempt could then be made at the challenge of compensating for different sequencing protocols.

Transcriptomic sequencing data could give a valuable additional perspective to this study. Such information, showing which genes are actively being transcribed, might give a more reliable functional perspective than metagenomic data. For example, archaea have been shown to have larger transcriptomic activity than would be expected based upon their (metagenomic) abundance (Wirth et al. 2023). While the potential of metatranscriptomics is great, however, handling RNA is more demanding than both 16S rRNA amplicon sequencing and metagenomics. Implementing RNA-based monitoring in biogas processes would therefore likely be more challenging than implementing DNA-based monitoring. Other possible workflows for generating sequencing count data include assembly-free annotation tools, or 16S rRNA-based functional annotation tools. We do not, however, expect such changes in the sequence processing to alter the conclusions of our study.

### Power, design, and stability

While our results do not support our hypothesis, they cannot conclusively dismiss it. Even though this study included multiple independent data sets, the number of independent reactors is still too small for a statistical test with sufficient power to compare the results of the functional and taxonomic count types. The multiple hierarchy levels for each count type further increased the number of features relative to the sample size. A promising analytic alternative would be to apply bootstrapping, which allows for the estimation of confidence intervals for complex metrics (Hastie et al. 2016). An example of such an application would be estimating the size of the best-performing model per count type for every study. Just like other statistical methods however, bootstrapping is sensitive to sample size.

When reflecting on the studies and samples that were included in the presented analysis, the Ahrens study stands out. Its reactor parameters look different compared to the other studies, having an inhibition of methane production that was only partial as well as a quick recovery of VFA levels. Furthermore, the first PCs of all count types of the Ahrens study indicate that reactors had not stabilized after inoculation; this start-up effect overlapped with the disturbance status classification. Omitting this variable from logistic regression should therefore cause a bias in its output, but using the study Ahrens for training or testing did not give observably lower performance than the other studies. Including a control reactor might have more clearly separated stabilization from disturbance effects, as was visible in the first PC of the Lemaigre study.

The general poor cross-study testing performance, together with the lack of shared features selected by glmnet, show that models are unstable in their feature selection. One reason for this could be that models were selected for having small feature size at their optimal performance, instead of accepting larger models with potentially lower variance, as recommended by Friedman et al. (2010). Besides accepting larger models, iterative approaches such as stability selection (Meinshausen and Bühlmann 2010) and the integration of feature selection across studies could be attempted.

### Implications for biogas production

The background of this study lies in the relevance of predicting disturbances in biogas plants; an expensive risk in the production of biogas. The goal of looking into functional and taxonomic data of microbial communities is to identify signals that warn for a disturbance even earlier than process parameters such as VFA. Alternatively the functional and taxonomic perspectives could give an estimate of a reactor’s sensitivity to disturbance, thereby allowing stable reactors to be run at their full organic loading rate capacity, which in turn increases gas production. A recent example of using microbial data to better understand reactor dysfunction is the unravelling of foaming in the anaerobic digestion of sewage sludge by Krohn et al. (2025). In the current study a sequencing protocol based around Illumina technology was used, which has long turnaround times, and that could delay feedback of information in an application at biogas plants. A modification in the protocol that could improve throughput would be the implementation of portable sequencing technologies, such as those using nanopores.

Although functional metagenomics offers theoretical advantages, such as more fine-grained modelling and reduced redundancy, its performance in this study was inconsistent. Functional metagenomics did not clearly outperform taxonomic metagenomics, which in turn did not consistently perform better than 16S rRNA amplicon sequencing. Based on these results, metagenomic sequencing appears to provide limited added value for routine monitoring of biogas systems compared to amplicon sequencing.

## Supplementary Material

fiag029_Supplemental_Files

## Data Availability

Raw sequencing reads have been deposited in the European Nucleotide Archive at EMBL-EBI under accession number PRJEB91257. The annotated features and gene counts, which are the starting point of the statistical analysis, have been deposited with relevant metadata in Zenodo (https://doi.org/10.5281/zenodo.18503758). The adapted version of the nbis-meta workflow for sequence processing has been shared through the GitHub account Dries-B as repository functional-vs-taxonomic-metagenomics--nbis-meta. The code for statistic analysis was shared as repository functional-vs-taxonomic-metagenomics--analysis and the code for cross-study testing separately as repository functional-vs-taxonomic-metagenomics--analysis--cross-study-testing. The code was also deposited in Zenodo; the sequence processing workflow at https://doi.org/10.5281/zenodo.18506251 and the combined code for statistic analysis and cross-study testing combined at https://doi.org/10.5281/zenodo.18506782.
