# Supplementary material for: Comparing the performance of functional versus taxonomic metagenomics for detecting ammonia disturbances in the biogas system
Source: FEMS Microbiol Ecol. 2026 Mar 20;102(5):fiag029. doi: 10.1093/femsec/fiag029 (PMC13098368; doi:10.1093/femsec/fiag029)
Supplement: fiag029_Supplemental_Files [file fiag029_supplemental_files.zip › supplementary data - file 1 - hypothesis and prediction.pdf]

Hypotheses and predictions

Page:	**Hypotheses and predictions**
Experiment:	**General comparison of functional vs taxonomic metagenomics during ammonia stress**
Book:	**Functional metagenomics in the biogas system**

ELN-P-3073-

Created by: Dries Boers (2022-09-09 11:37:41)
Modified by: Dries Boers (2022-09-09 12:09:48)

Page: **Hypotheses and predictions**

Created by: Dries Boers (2022-09-09 11:37:41)

Modified by: Dries Boers (2022-09-09 12:09:48)

ELN-P-3073-

Content

The hypotheses and predictions below were defined before sequencing datasets from LIST institute and Chapleur group were downloaded from Eurofins servers.

Content

Hypothesis 1: Functional metagenomics represents the biogas process better than taxonomic metagenomics.

> Prediction 1.1: When comparing different replicate reactors for a timepoint, functional metagenomic data will have a smaller variance compared to taxonomic metagenomic data.

Signature page

This document has been electronically signed using eduSign.

eduSign

Electronically signed by
Anna Schnürer

Date and time of signature
2022-11-01 14:31 UTC

Authenticated by
Sveriges Lantbruksuniversitet

Validate signed documents at: https://validator.edusign.sunet.se

SUNET
