# Supplementary material for: Comparing the performance of functional versus taxonomic metagenomics for detecting ammonia disturbances in the biogas system
Source: FEMS Microbiol Ecol. 2026 Mar 20;102(5):fiag029. doi: 10.1093/femsec/fiag029 (PMC13098368; doi:10.1093/femsec/fiag029)
Supplement: fiag029_Supplemental_Files [file fiag029_supplemental_files.zip › supplementary data - file 2 - statistical analysis.Rmd.pdf]

Regularized regression

```r
library(glmnet)
library(vegan)

library(stringr)
library(tidyr)
library(knitr)
library(purrr)
library(dplyr)
library(magrittr)

source("0_data_wrangling/functions/sample_names_to_dataframe_f.R")

### Load data -----------------------------------------------------------------------------------------

counts_m_l <- c(readRDS("0_data_wrangling/counts__16S.rds") %>%
                  { set_names(., paste0('16S_', names(.)) ) },
                readRDS("0_data_wrangling/taxonomic/counts__metagenomics.rds") %>%
                  { set_names(., paste0('GAT_', names(.)) ) },
                readRDS("0_data_wrangling/functional/counts__metagenomics.rds")
                )

samples_disturbance_status_fct <- readRDS("0_data_wrangling/sample_disturbance_status_fct.rds")


## Only keep samples of interest
counts_selected__m_l <- counts_m_l %>%
    lapply(function(m) { m[names(samples_disturbance_status_fct), ] } )

### Transform data ------------------------------------------------------------------------------------

counts_selected_clr__m_l <- counts_selected__m_l %>%
    lapply( function(m) { decostand(m, method='clr', MARGIN=1, pseudocount=1) } )

# ## Standardization: performed by glmnet

### Regularized regression ---------------------------------------------------------------------------

## For alpha, the elastic net mixing parameter, a value of 0.5 is used.
## This is used to balance the advantages and disadvantages of
## lasso regression (alpha = 1) and ridge regression (alpha = 0).

## Custom, low values are used for `lambda=`, because default values (down to 10^-2) do not show the optimum.
## First iteration (iter1) is used to determine maximum lambda.

foldid_v_l <- counts_selected_clr__m_l %>%
    lapply(rownames) %>%
    lapply(function(names) {
        names %>%
            sample_names_to_dataframe_f() %>%
            { .$reactor } %>% setNames(names) %>%
            as.factor() %>% as.numeric() } )

foldid_v_l %>% { if (any(!duplicated(.) & !duplicated(., fromLast=TRUE) ) ) {
  warning("FoldIDs not identical for all data types.") } }

### Iteration 1: default lambdas
mapply(
    FUN = function(m, count_type, foldid_v) {
        cv.glmnet(x=m,
                  y=samples_disturbance_status_fct[rownames(m) ],
                  family='binomial',
                  type.measure='default',
                  alpha=0.5,
                  standardize=TRUE,
                  #> Default. Also recommended in https://stats.stackexchange.com/questions/86434.
                  #> It would be good to favour high-count features,
                  #> because they will be less affected by the pseudocount of CLR,
                  #> but I have not found a standard way of doing this.
                  foldid = foldid_v,
                  trace.it=1 ) %>%
            saveRDS(paste0("glmnet__logistic.R.o/", count_type, "__iter1.rds" ) ) },

    m=counts_selected_clr__m_l,
    count_type=names(counts_selected_clr__m_l),
    foldid_v=foldid_v_l,
    SIMPLIFY=FALSE) %>%
    invisible()



glmnet__iter1__min_lambda__l <-
    list.files("glmnet__logistic.R.o/", pattern='*') %>%
    setNames(., str_remove(., "__iter1.rds" ) ) %>%
    ## Reorder
    .[names(counts_selected__m_l) ] %>%
    lapply( function(filename) { readRDS( paste0("glmnet__logistic.R.o/", filename) ) } ) %>%
    lapply( function(glmnet_l) { min( glmnet_l$lambda ) } )

# ------------------------------------------------------------------------------------------------------

# divide_by_abs_f <- function(v) { v / abs(v) }
get_sign_f <- function(v) { v / abs(v) }

generate_lambdas_f <-
  function(start_lambda, end_lambda = .Machine$double.xmin, nlambda=100) {
    log_start <- log10(start_lambda)
    log_end <- log10(end_lambda)
    log_vec <- seq(from= get_sign_f(log_start) * log2(abs(log_start)),
                   to=   get_sign_f(log_end) * log2(abs(log_end)),
                   length.out=nlambda-1)
    c(10^( get_sign_f(log_vec) * 2^abs(log_vec) ),
      0)
  }

### Iteration 2: to minimal lambda
mapply(
    FUN = function(m, count_type, foldid_v, min_lambda__iter1) {
        cv.glmnet(x=m,
                  y=samples_disturbance_status_fct[rownames(m) ],
                  family='binomial',
                  type.measure='default',
                  alpha=0.5,
                  lambda=generate_lambdas_f( start_lambda = min_lambda__iter1, nlambda = 50 ),
                  #> Lambda decreases to 0 (no penalization -> full model).
                  #> Decreasing order allows glmnet to start run at near-optimal value ('warm start').
                  #> Using .Machine$double.xmin allows getting closest values to 1 and 0.
                  standardize=TRUE,
                  foldid = foldid_v,
                  maxit=5000,
                  trace.it=1 ) %>%
            saveRDS(paste0("glmnet__logistic.R.o/", count_type, "__iter2.rds") ) },

    m=counts_selected_clr__m_l,
    count_type=names(counts_selected_clr__m_l),
    foldid_v=foldid_v_l,
    min_lambda__iter1=glmnet__iter1__min_lambda__l,
    SIMPLIFY=FALSE) %>%
    invisible()
#> "Warning: one multinomial or binomial class has fewer than 8  observations; dangerous ground"
#> #> Warning above is given for multiple folds.
#> "Warning: collapsing to unique 'x' values"
#> #> Warning above is given many times (probably each fold), only for second iteration (iter2).
#> "Warning: Option grouped=FALSE enforced in cv.glmnet, since < 3 observations per fold"
#> #> Warning above is given per study.
#>
#> The latter two warnings can be disregarded.
#> The first warning shows a challenge in working with this dataset,
#> but it is circumvented when data of multiple studies is combined,
#> and in any case, regularized regression is the most suitable analytic framework

sessionInfo()
```

```
#> R version 4.4.0 (2024-04-24)
#> Platform: x86_64-pc-linux-gnu
#> Running under: Linux Mint 21.2
#>
#> Matrix products: default
#> BLAS:   /usr/lib/x86_64-linux-gnu/blas/libblas.so.3.10.0
#> LAPACK: /usr/lib/x86_64-linux-gnu/lapack/liblapack.so.3.10.0
#>
#> locale:
#>  [1] LC_CTYPE=en_US.UTF-8       LC_NUMERIC=C               LC_TIME=sv_SE.UTF-8        LC_COLLATE=en_US.UTF-8
LC_MONETARY=sv_SE.UTF-8
#>  [6] LC_MESSAGES=en_US.UTF-8    LC_PAPER=sv_SE.UTF-8       LC_NAME=C                  LC_ADDRESS=C
LC_TELEPHONE=C
#> [11] LC_MEASUREMENT=sv_SE.UTF-8 LC_IDENTIFICATION=C
#>
#> time zone: Europe/Stockholm
#> tzcode source: system (glibc)
#>
#> attached base packages:
#> [1] stats     graphics  grDevices utils     datasets  methods   base
#>
#> other attached packages:
#>  [1] magrittr_2.0.3 dplyr_1.1.4    purrr_1.0.2    knitr_1.46     tidyr_1.3.1    stringr_1.5.1  vegan_2.6-4    lat
tice_0.22-6 permute_0.9-7  glmnet_4.1-8
#> [11] Matrix_1.7-0
#>
#> loaded via a namespace (and not attached):
#>  [1] highr_0.10        compiler_4.4.0    tidyselect_1.2.1  Rcpp_1.0.12       parallel_4.4.0    cluster_2.1.6
splines_4.4.0     R6_2.5.1
#>  [9] generics_0.1.3    shape_1.4.6.1     iterators_1.0.14  MASS_7.3-60.2     tibble_3.2.1      pillar_1.9.0
rlang_1.1.3       utf8_1.2.4
#> [17] stringi_1.8.3     xfun_0.43         cli_3.6.2         mgcv_1.9-1        foreach_1.5.2     grid_4.4.0
rstudioapi_0.16.0 lifecycle_1.0.4
#> [25] nlme_3.1-164      vctrs_0.6.5       evaluate_0.23     glue_1.7.0        codetools_0.2-20  survival_3.6-4
fansi_1.0.6       tools_4.4.0
#> [33] pkgconfig_2.0.3
```

Selection of optimal hierarchy level per count type

```r
library(plotly)
library(cowplot)
library(broom)
library(tidyverse)
library(magrittr)

source("glmnet_plot__functions/plot_f.R", chdir=TRUE)
#> Includes filter on regression type
```

Load data

```r
df <-
    c('logistic','linear') %>%
    set_names(., value = .) %>%
    lapply( function(str) { str %>%
            paste0('glmnet__', ., ".R.o") %>%
            list.files(pattern ='*', full.names = TRUE) %>%
            setNames(nm = .) %>%
            lapply(function(path) { path %>%
                    readRDS() %>%
                    tidy() } ) %>%
            bind_rows(.id = 'path') } ) %>%
    bind_rows(.id = "regression_type")

df <- df %>%
    mutate(iteration = str_extract(path, "iter[12]"), .after=regression_type) %>%
    mutate(count_type =
               str_remove(path, "glmnet.*/") %>%
               str_remove("__iter[12].rds"),
           .keep='unused', .after=regression_type)
```

Selection of optimal hierarchy level per count type

As stated in Supplementary Figure 1: “Count types with low loss scores with a small number of included variables are selected.”

In the case of similar performance, higher level count types are preferred, as they are expected to perform better in cross-study testing. Because of the same reason (cross-study testing), a higher level count type is included when OTU/ASV or ‘as is’ performs best in 16S and GAT, respectively.

16S

```r
`16S_p` <-
    plot_grid(plot_f('logistic', filter(df, startsWith(count_type, "16S") ) ),
              plot_f('linear', filter(df, startsWith(count_type, "16S") ) ) )
    #> Separately add legend in grid?
    #> #> For now, this is not worth it.
print(`16S_p`)
```

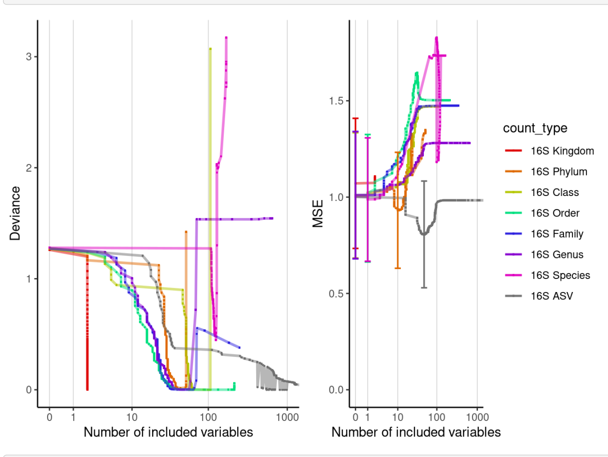


```r
df <- filter(df, ( regression_type == 'logistic' &
                       ( count_type %in% "16S_Order" | !startsWith(count_type, '16S') ) ) |
                 ( regression_type == 'linear' &
                       ( count_type %in% "16S_as_is" | !startsWith(count_type, '16S') ) ) )
```

In logistic regression, order level performs best, while in linear regression, ‘as is’ performs best (but its +1SE range overlaps somewhat with other count_types).

GAT: Gene-level annotation of taxonomy

```r
GAT_p <-
    plot_grid(
        plot_f('logistic',filter(df, startsWith(count_type, 'GAT') ) ),
        plot_f('linear', filter(df, startsWith(count_type, 'GAT') ) ) )
print(GAT_p)
```

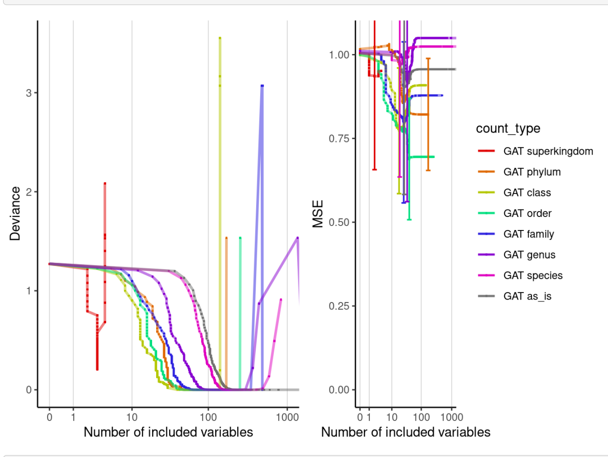


```r
df <- filter(df, ( regression_type == 'logistic' &
                     ( count_type %in% "GAT_class" ) | !startsWith(count_type, 'GAT') ) |
                 ( regression_type == 'linear' &
                     ( count_type %in% "GAT_order" | !startsWith(count_type, 'GAT') ) ) )
```

Functional

```r
functional_p <-
    plot_grid(plot_f('logistic', filter(df, startsWith(count_type, 'eggNOG') |
                                            startsWith(count_type, 'kegg') ) ),
              plot_f('linear', filter(df, startsWith(count_type, 'eggNOG') |
                                          startsWith(count_type, 'kegg') ) ) )
print(functional_p)
```

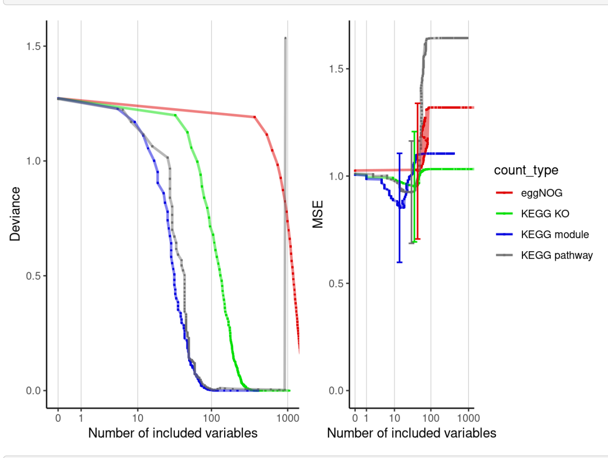


```r
df <- filter(df, ( regression_type == 'logistic' &
                     ( count_type %in% "kegg_pathway" | !startsWith(count_type, 'kegg') ) ) |
                 ( regression_type == 'linear' &
                     ( count_type %in% "kegg_module" | !startsWith(count_type, 'kegg') ) ) )
#> eggNOG does not need to be filtered, because there is only a single type.
```

```r
all_count_types__grid__p <-
    plot_grid(plotlist=list(`16S_p`,
                            GAT_p,
                            functional_p),
              ncol=1)
print(all_count_types__grid__p)
```

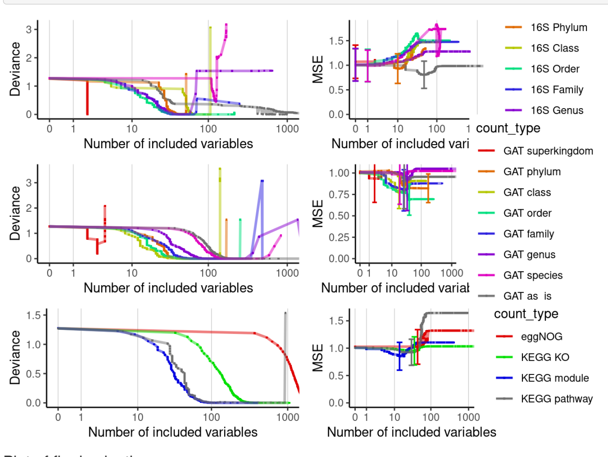

Plot of final selection

```r
plot_grid(plot_f('logistic', df) + theme(legend.position='right'),
          plot_f('linear', df) )
```

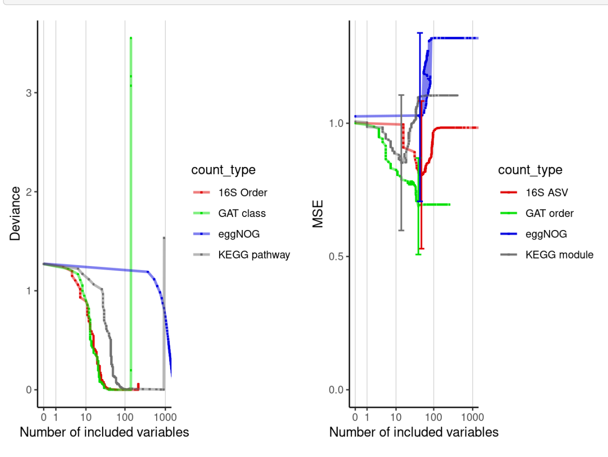

Identify smallest logistic models with low loss

For logistic glmnet, the +1SE rule does not seem to work, because minimal deviance estimates are so low that their standard error becomes 0. So instead, look for the smallest models before deviance starts increasing.

```r
smallest_low_loss_models_df_l <- list()

p_l <- readRDS("glmnet_plot__data_selection__df.rds") %>%
    filter(regression_type == 'logistic') %>%
    { split(., f = .$count_type) } %>%
    { mapply(
        df = .,
        count_type = names(.),
        FUN =
            function(df, count_type) {
                ggplot(data = df,
                       mapping=aes(x=log10(nzero+1) ) ) +
                    geom_point(mapping=aes(y=estimate),
                               shape='square',
                               size=0.5,
                               alpha=3/4) +
                    geom_line(mapping=aes(y=estimate),
                              linewidth = 1,
                              alpha=1/2
                              #> Prevent oversaturated colours.
                    ) +

                    coord_cartesian(xlim = c(log10(0+1), log10(1000+1)),
                                    ylim = c(0, max( df %>% filter(nzero <= 1000) %>% .$estimate) ) ) +
                    scale_x_continuous(breaks = c(0,1,10,100,1000) %>% { log10(.+1) },
                                       labels = c(0,1,10,100,1000) ) +
                    xlab("Number of included variables") +
                    ylab("Deviance") +
                    ggtitle(count_type) +
                    theme_classic() +
                    theme(panel.grid.major.x=element_line(color = "gray80", linewidth = 0.25) )
            },
        SIMPLIFY = FALSE ) }

p_l %>%
    lapply(ggplotly)
ggplotly(p_l$eggNOG +
    coord_cartesian(xlim = c(log10(0+1), log10(10000+1)),
                    ylim = c(0, max( df %>% filter(nzero <= 1000) %>% .$estimate) ) ) )



logistic__smallest_low_loss_models_df_l <-
    ## Values found using ggplotly, with an inverse pseudolog transformation.
    list(`16S_Order` = 10^1.6127839 - 1,
         GAT_class = 10^1.6127839 - 1,
         eggNOG = 10^3.287354 - 1,
         kegg_pathway = 10^2.0413927 - 1) %>%
    stack() %>%
    set_colnames(c('n_params','count_type'))

# saveRDS(logistic__smallest_low_loss_models_df_l, "glmnet__logistic__smallest_low_loss_models.rds")
```

```r
sessionInfo()
```

```
#> R version 4.4.0 (2024-04-24)
#> Platform: x86_64-pc-linux-gnu
#> Running under: Linux Mint 21.2
#>
#> Matrix products: default
#> BLAS:   /usr/lib/x86_64-linux-gnu/blas/libblas.so.3.10.0
#> LAPACK: /usr/lib/x86_64-linux-gnu/lapack/liblapack.so.3.10.0
#>
#> locale:
#>  [1] LC_CTYPE=en_US.UTF-8       LC_NUMERIC=C               LC_TIME=sv_SE.UTF-8        LC_COLLATE=en_US.UTF-8
#>  [5] LC_MONETARY=sv_SE.UTF-8    LC_MESSAGES=en_US.UTF-8    LC_PAPER=sv_SE.UTF-8       LC_NAME=C
#>  [9] LC_ADDRESS=C               LC_TELEPHONE=C             LC_MEASUREMENT=sv_SE.UTF-8 LC_IDENTIFICATION=C
#>
#> time zone: Europe/Stockholm
#> tzcode source: system (glibc)
#>
#> attached base packages:
#> [1] stats     graphics  grDevices utils     datasets  methods   base
#>
#> other attached packages:
#>  [1] magrittr_2.0.3  lubridate_1.9.3 forcats_1.0.0   stringr_1.5.1   dplyr_1.1.4     purrr_1.0.2     tidyr_1.3.1
tibble_3.2.1
#>  [9] tidyverse_2.0.0 broom_1.0.5     cowplot_1.1.3   plotly_4.10.4   ggplot2_3.5.1   readr_2.1.5
#>
#> loaded via a namespace (and not attached):
#>  [1] sass_0.4.9        utf8_1.2.4        generics_0.1.3    stringi_1.8.3     hms_1.1.3         digest_0.6.35
timechange_0.3.0
#>  [8] evaluate_0.23     grid_4.4.0        fastmap_1.1.1     jsonlite_1.8.9    backports_1.4.1   httr_1.4.7
fansi_1.0.6
#> [15] viridisLite_0.4.2 scales_1.3.0      lazyeval_0.2.2    jquerylib_0.1.4   cli_3.6.2         rlang_1.1.3
crayon_1.5.2
#> [22] bit64_4.0.5       munsell_0.5.1     withr_3.0.0       cachem_1.0.8      tools_4.4.0       parallel_4.4.0
tzdb_0.4.0
#> [29] colorspace_2.1-0  vctrs_0.6.5       R6_2.5.1          lifecycle_1.0.4   htmlwidgets_1.6.4 bit_4.0.5
vroom_1.6.5
#> [36] pkgconfig_2.0.3   pillar_1.9.0      bslib_0.7.0       gtable_0.3.5      glue_1.7.0        data.table_1.15.4
highr_0.10
#> [43] xfun_0.43         tidyselect_1.2.1  rstudioapi_0.16.0 knitr_1.46        farver_2.1.1      htmltools_0.5.8.1
labeling_0.4.3
#> [50] rmarkdown_2.26    compiler_4.4.0
```
