# Supplementary material for: Comparing the performance of functional versus taxonomic metagenomics for detecting ammonia disturbances in the biogas system
Source: FEMS Microbiol Ecol. 2026 Mar 20;102(5):fiag029. doi: 10.1093/femsec/fiag029 (PMC13098368; doi:10.1093/femsec/fiag029)
Supplement: fiag029_Supplemental_Files [file fiag029_supplemental_files.zip › Supplementary Tables.docx]

Supplementary Table 1. Feeding phases for Cardona and Lemaigre studies.

|  | **Feed type** | **Day 1-70** | **Day 70-84** | **Day 84-129a** | **Day 129-231** |  |
| --- | --- | --- | --- | --- | --- | --- |
| **Cardona study** | **Food biowaste (gCOD/L/day)** | 0.5 | 0.5 | 0 | 0.5 |  |
|  | **Nitrogen: ammonia (g/L/day)** | 0 | 4b | 0 | 2 |  |
|  |  |  |  |  |  |  |
|  | **Feed type** | **Week 1-8** | **Week 8-19** | **Week 20-22** | **Week 23-26** | **Week 27-34** |
| **Lemaigre studyc,d** | **Carbon: beet pulp (gVS/L/week)** | 10-13e | 10-13 | 10-13 | 0 | 8-13e |
|  | **Nitrogen: urea (g/L/week)** | 0 | 1-4f | 8-10f | 0 | 0 |

VS: volatile solids.

a. Feeding and reactor flowthrough was fully stopped during this phase.
b. Ammonia was added “abruptly” to reach a concentration of 4 g/L within the day.
c. Feeding varied per week within ranges.
d. Control reactor was fed constantly with 8-13 gVS/L/week beet pulp.
e. Feeding was built from lower values in first two weeks of phase.
f. Urea feeding was gradually increased during phases.

Supplementary Table 2. Mean ± standard deviance across samples of number of reads, and percentages remaining after steps of sequence processing.

|  | **raw reads (millions)** | **trimming (%)** | **assembly (%)** | **mapping to predicted genes**  **(%)** |
| --- | --- | --- | --- | --- |
| Lemaigre study | 12.0 ± 2.6 | 96.6 ± 0.4 | 87.1 ± 1.1 | 61.0 ± 8.0 |
| Cardona study | 11.7 ± 2.8 | 94.1 ± 2.2 | 84.4 ± 2.3 | 65.5 ± 3.1 |
| Ahrens study | 12.2 ± 1.5 | 95.2 ± 0.7 | 83.8 ± 1.0 | 67.2 ± 2.3 |

Supplementary Table 3. Mean ± standard deviance across samples of annotated counts of different count types at different hierarchical levels. GAT: gene-level annotation of taxonomy.

|  | **total counts**a **(millions)** | **GAT (%)** | **GAT species (%)** | **eggNOG (%)** | **KEGG KO (%)** | **KEGG**  **module**b **(%)** |
| --- | --- | --- | --- | --- | --- | --- |
| Lemaigre study | 9.42 ± 3.01 | 93.9 ± 1.8 | 6.55 ± 2.23 | 89.8 ± 3.3 | 57.9 ± 2.8 | 26.0 ± 1.3 |
| Cardona study | 9.48 ± 2.56 | 96.0 ± 0.9 | 8.65 ± 1.17 | 94.2 ± 1.2 | 65.2 ± 1.9 | 28.4 ± 0.8 |
| Ahrens study | 10.2 ± 1.2 | 96.4 ± 0.6 | 5.66 ± 1.56 | 91.8 ± 1.1 | 60.2 ± 1.6 | 25.7 ± 0.8 |

a. Note that the total count is larger than the number of reads mapping to a gene (Supplementary Table 2), because reads can overlap multiple genes.

b. As a KEGG KO can be part of multiple KEGG modules, the number of counts of KEGG module can be inflated.

Supplementary Table 4. Optimal hierarchy levels per study, regression type and count type. eggNOG is omitted, because this data does not have multiple hierarchy levels. GAT: gene-level annotation of taxonomy.

| **Study** | **Regression type** | **Count type** | **Optimal hierarchy level** |
| --- | --- | --- | --- |
| Lemaigre | logistic | 16S | phylum |
|  |  | GAT | phylum |
|  |  | KEGG | module |
|  | linear | 16S | OTU |
|  |  | GAT | class |
|  |  | KEGG | pathway |
| Cardona | logistic | 16S | phylum |
|  |  | GAT | phylum |
|  |  | KEGG | module |
|  | linear | 16S | genus |
|  |  | GAT | genus |
|  |  | KEGG | module |
| Ahrens | logistic | 16S | order |
|  |  | GAT | class |
|  |  | KEGG | pathway |
|  | linear | 16S | ASV |
|  |  | GAT | order |
|  |  | KEGG | module |

Supplementary Table 5. Own-study performance of trained regularised regression models. The ‘AUC / R2 ’ and ‘Deviance / MSE’ columns represent AUC and Deviance for logistic regression and R2  and MSE for linear regression, respectively. Cell shading has been added per column. AUC: area under the receiver operating characteristic curve. GAT: gene-level annotation of taxonomy. MSE: mean squared error. R2: the coefficient of determination.

Supplementary Table 6. Number of shared extracted features between logistic and linear regression, per count type and study. Count types overlapping at different hierarchical levels (for example, a species with its genus) was taken into account for 16S, GAT and eggNOG. GAT: gene-level annotation of taxonomy.

|  |  | **16S** | **GAT** | **eggNOG** | **KEGG** |
| --- | --- | --- | --- | --- | --- |
| **Lemaigre study** |  | 19/51 | 6/64 | 16/1932 | 0/143a |
| **Cardona study** |  | 29/46 | 80/129 | 59/1683 | 61/485 |
| **Ahrens study** |  | 22/88 | 12/80 | 25/2213 | 0/123a |

a. Shared features between KEGG count types are missed when they are at different hierarchical levels, such as module and pathway.

Supplementary Table 7. Number of shared extracted features between all studies, per count type and regression type. Count types overlapping at different hierarchical levels (for example, a species with its genus) was taken into account for 16S, GAT and eggNOG. GAT: gene-level annotation of taxonomy.

|  |  | **16S** | **GAT** | **eggNOG** | **KEGG** |
| --- | --- | --- | --- | --- | --- |
| **Logistic regression** |  | 33/86 | 36/122 | 86/4463 | 10/281a |
| **Linear regression** |  | 1/99 | 18/151 | 70/1365 | 13/470a |

a. Shared features between KEGG count types are missed when they are at different hierarchical levels, such as module and pathway.
