# Supplementary figures and images for: Comparing the performance of functional versus taxonomic metagenomics for detecting ammonia disturbances in the biogas system
Source: FEMS Microbiol Ecol. 2026 Mar 20;102(5):fiag029. doi: 10.1093/femsec/fiag029 (PMC13098368; doi:10.1093/femsec/fiag029)

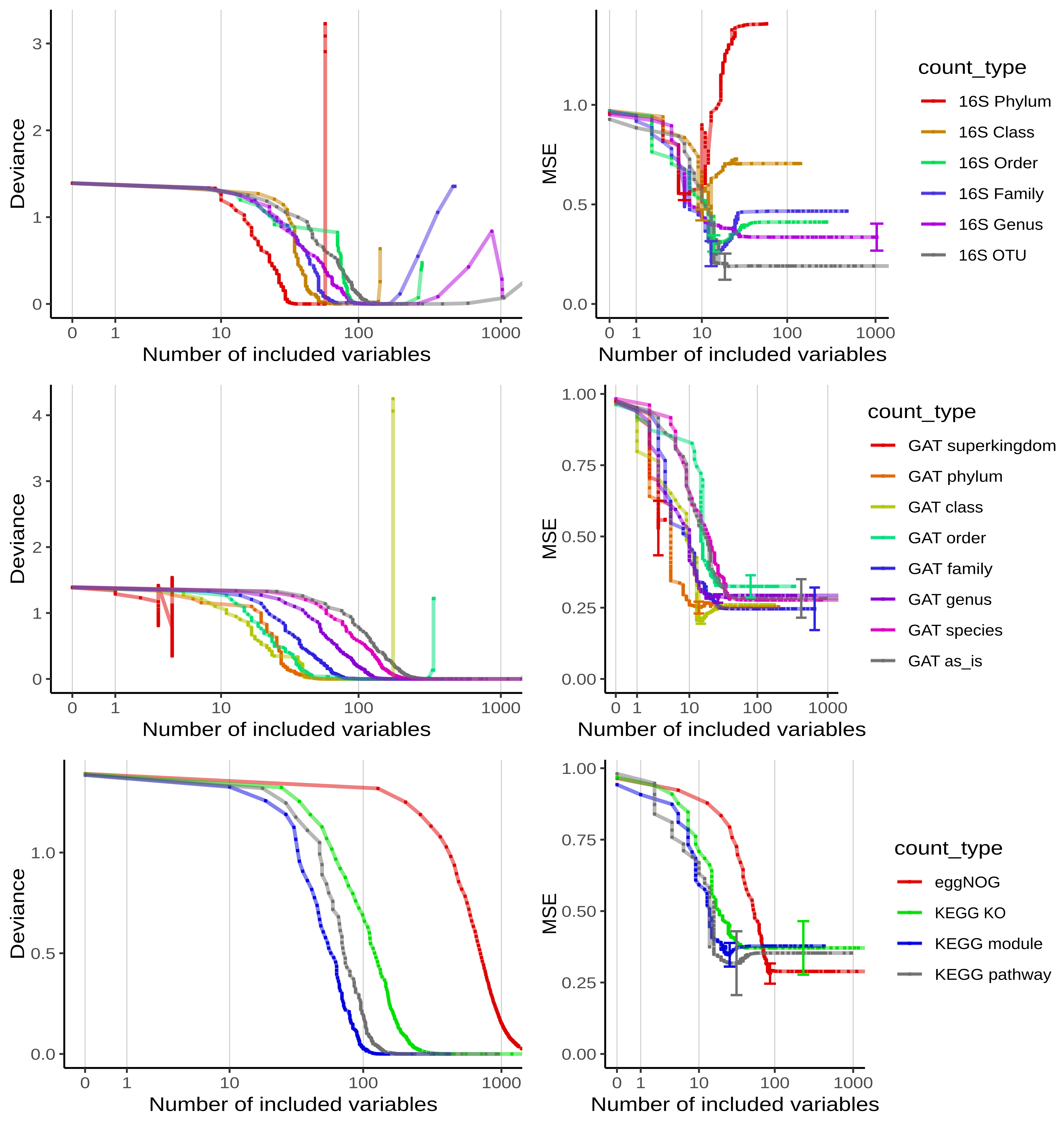

Supplement: fiag029_Supplemental_Files [file fiag029_supplemental_files.zip › supplementary figure 1.png]

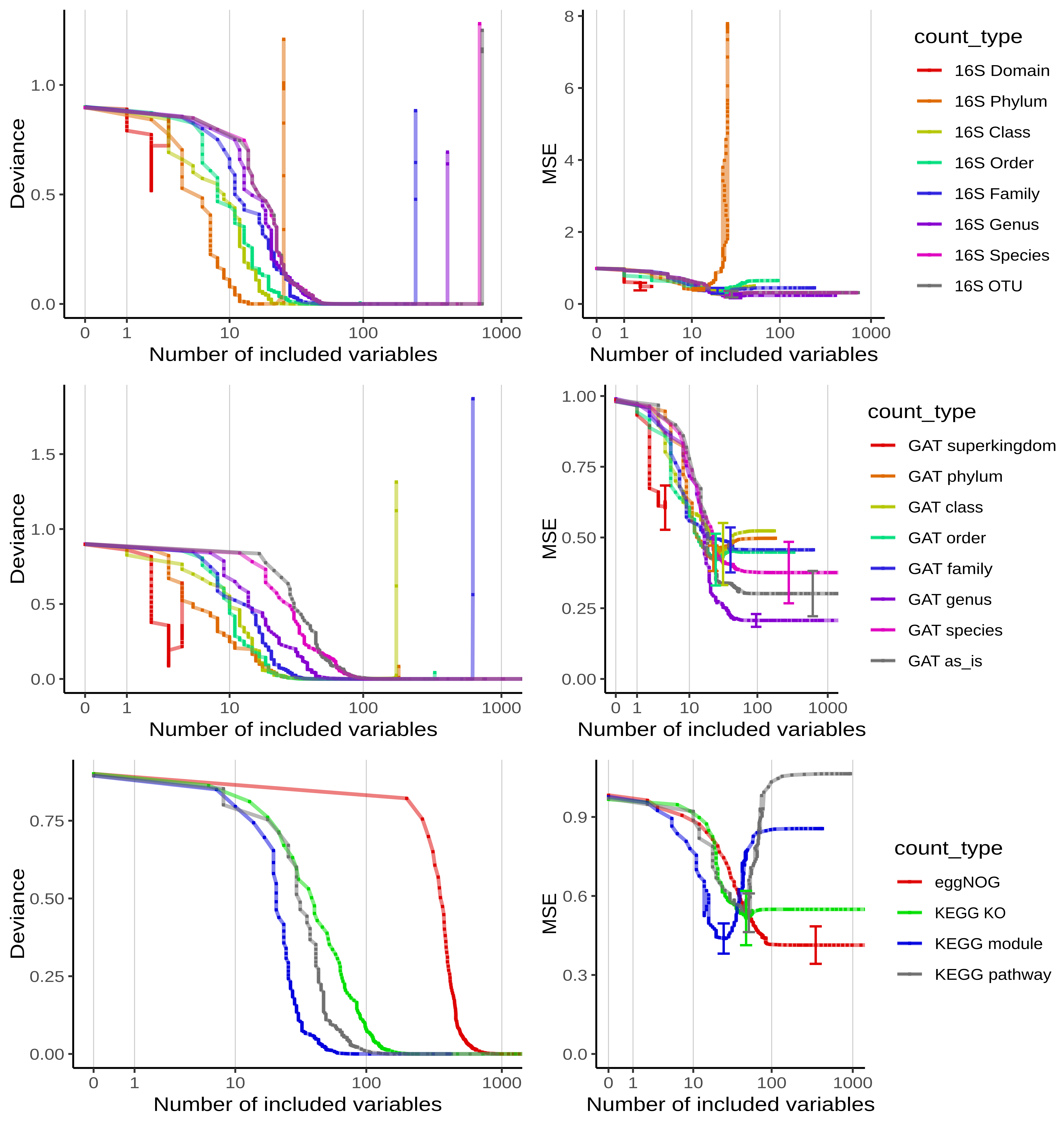

Supplement: fiag029_Supplemental_Files [file fiag029_supplemental_files.zip › supplementary figure 2.png]

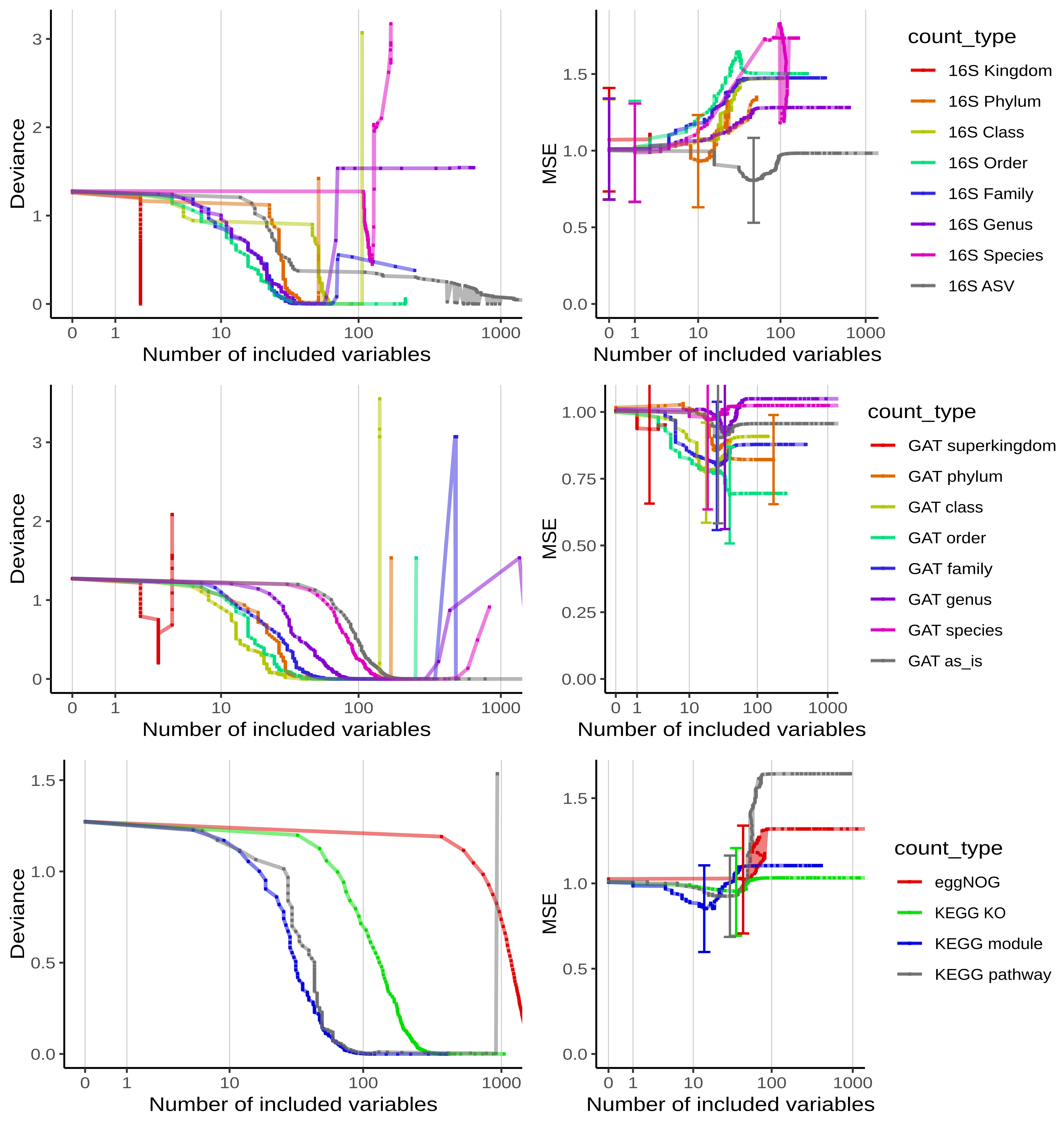

Supplement: fiag029_Supplemental_Files [file fiag029_supplemental_files.zip › supplementary figure 3.png]
